# The High Burden of Cholera in Children: Comparison of Incidence from Endemic Areas in Asia and Africa

**DOI:** 10.1371/journal.pntd.0000173

**Published:** 2008-02-20

**Authors:** Jacqueline L. Deen, Lorenz von Seidlein, Dipika Sur, Magdarina Agtini, Marcelino E. S. Lucas, Anna Lena Lopez, Deok Ryun Kim, Mohammad Ali, John D. Clemens

**Affiliations:** 1 International Vaccine Institute, Seoul, Korea; 2 National Institute of Cholera and Enteric Diseases, Kolkata, India; 3 National Institute of Health Research and Development, Jakarta, Indonesia; 4 Ministry of Health, Maputo, Mozambique; National Institute of Allergy and Infectious Diseases, United States of America

## Abstract

**Background:**

Cholera remains an important public health problem. Yet there are few reliable population-based estimates of laboratory-confirmed cholera incidence in endemic areas around the world.

**Methods:**

We established treatment facility–based cholera surveillance in three sites in Jakarta (Indonesia), Kolkata (India), and Beira (Mozambique). The annual incidence of cholera was estimated using the population census as the denominator and the age-specific number of cholera cases among the study cohort as the numerator.

**Findings:**

The lowest overall rate was found in Jakarta, where the estimated incidence was 0.5/1000 population/year. The incidence was three times higher in Kolkata (1.6/1000/year) and eight times higher in Beira (4.0/1000/year). In all study sites, the greatest burden was in children under 5 years of age.

**Conclusion:**

There are considerable differences in cholera incidence across these endemic areas but in all sites, children are the most affected. The study site in Africa had the highest cholera incidence consistent with a growing impression of the large cholera burden in Africa. Burden estimates are useful when considering where and among whom interventions such as vaccination would be most needed.

## Author Summary

Cholera is an often forgotten disease affecting the world's forgotten people. When a large cholera outbreak occurs, the disease appears briefly on the radar of public attention. Some unfortunate populations around the world suffer recurrent episodes of cholera but their plight goes unnoticed. We established cholera surveillance in impoverished areas in Jakarta (Indonesia), Kolkata (India), and Beira (Mozambique) where the disease is known to occur regularly. The cholera burden was calculated using the site population as the denominator and the number of cholera cases as the numerator. The lowest overall rate was in Jakarta with 0.5 cases per 1000 population per year. The incidence was three times higher in Kolkata (1.6/1000/year) and eight times higher in Beira (4.0/1000/year), adding to the growing impression of the large cholera problem in Africa. In all sites, children are the most affected. Estimates such as these are useful when considering where and among whom interventions against the disease are most needed. Improvement of water supply and sanitation is the best strategy against cholera and other diarrheal diseases but may not be achievable in these impoverished areas in the near future. Other immediate, short- to medium-term strategies such as vaccination against cholera may be useful.

## Introduction

Cholera is an acute, diarrheal illness caused by infection of the intestine with O1 or O139 serogroups of *Vibrio cholerae* [1]. Profuse watery diarrhea and vomiting can lead to dehydration and shock. Without treatment, death can occur within hours. Oral and intravenous rehydration therapy has markedly decreased case fatality rates [2],[3] but cholera remains a dreaded illness because of its rapid onset, severity, and potential to cause outbreaks that easily overwhelm public health systems in impoverished settings. Seasonal disease occurs in many less developed countries that cannot afford to establish or to maintain essential infrastructure for safe water supply and sanitation. Outbreaks may arise during natural disasters and complex emergencies.

In 2006, 52 countries officially reported a total of 236,896 cholera cases including 6,311 deaths with a CFR of 2.7%, to the World Health Organization (WHO) [4]. These numbers do not reflect the true burden of cholera due to limitations in the surveillance and notification systems of many countries where the disease is endemic, as well as widespread underreporting because of fear of unjustified travel and trade-related sanctions [4],[5]. Hospital-based studies and outbreak reports provide important information, but they usually do not have a clear population denominator to allow estimation of the age-specific incidence of cholera in a community. A further challenge in the estimation of disease burden is the dearth of microbiology laboratories capable of detecting *V. cholerae* O1 and O139. Reported cases are often based solely on clinical diagnosis of the illness, adding further uncertainty. In the recent past, population-based information on the burden of culture-confirmed cholera cases has come principally from a single research institution in Bangladesh [6].

Policymakers from several developing countries have indicated that information on the age-specific burden of cholera is essential to decide the urgency of control strategies, including vaccination [7]. We established population-based surveillance for cholera in three study areas in Indonesia, India, and Mozambique in preparation for potential vaccine trials [8]–[10]. The areas were selected based on known endemicity of cholera and the presence of pre-existing research infrastructure or the potential to create such infrastructure. Using similar methodology in the three study areas allowed us to compare the overall and age-specific incidence of cholera across sites. Various findings from the three sites have been published elsewhere [8]–[10]. In this article, we compare and contrast the incidence of cholera across the sites.

## Methods

### Study design

In each site, a catchment area was selected, census data were obtained, and surveillance for diarrhea was established in treatment centers serving the catchment population. A diarrhea episode was defined as passage of three or more loose or liquid stools in the 24 hour period prior to presentation for care. Repeat visits for the same episode of diarrhea, defined as three or fewer days apart between the end of the first episode and the onset of the second, were excluded. For every patient agreeing to participate, a case report form was completed and a rectal swab was obtained and inoculated into Cary-Blair transport media. The definition of diarrhea and laboratory methods was standardized across the study sites.

### Laboratory methods

Rectal swabs in Cary-Blair media were brought on the same day to the study laboratory and plated directly onto thiosulfate citrate bile salt sucrose (TCBS) agar (Eiken Chemical Company, Tokyo, Japan). The specimens were also incubated in alkaline peptone water (pH 8.6) for 6 to 8 hours at 37°C then plated onto TCBS. After overnight incubation at 37°C, suspected colonies on the TCBS plates were tested biochemically and confirmed by agglutination with polyvalent O1 and monovalent Ogawa and Inaba antisera (Difco Laboratories, Detroit, Michigan). Non-agglutinating strains were tested with antiserum to *V. cholerae* O139 strain.

Laboratory methods for isolation of *V. cholerae* were similar in each site. Isolation of *V. cholerae* was conducted in reference laboratories in two sites (the U.S. Naval Medical Research Unit No. 2 in Jakarta and the National Institute of Cholera and Enteric Diseases in Kolkata). In Beira, a consultant from the ICDDR,B: Centre for Health and Population Research, Dhaka, Bangladesh provided training and supervision to ensure standard procedures were followed. *V. cholerae* isolated in Beira were confirmed at the ICDDR,B.

### Sites

In Indonesia, the study area consisted of two adjacent districts (*kecamatans*), Tanjung Priok and Koja, in North Jakarta [8]. Residents live in homes that are temporary structures without running water and more than a third of households have no access to tap water. In 2001, the population in the catchment area was 160,257 (Table 1). Surveillance was conducted from August 2001 to July 2003 and included residents in the study site of all age groups who presented with diarrhea to participating health care providers: primary health centers (*puskesmas*) in Tanjung Priok and Koja, as well as the Infectious Disease Hospital and Koja Hospital. Rectal swabs were brought to the study laboratory for isolation of *V. cholerae* O1 and O139.

**Table 1:** Population, number of culture-positive cholera cases each year, and incidence (per 1000 population per year) in study sites in three countries.

Ages	Jakarta (August 2001 to July 2003)					Kolkata (1 May 2003 to 30 April 2005)					Beira** (December 2003 to January 2004)			
	Baseline population	Cases during first year	Cases during second year	Total cases	Annualized incidence	Mid-year population*	Cases during first year	Cases during second year	Total cases	Annualized incidence	Baseline population	Total cases	Incidence	Corrected incidence***
**<24 months**	6611	29	13	42	**3.2**	1562	19	8	27	**8.6**	not included in surveillance			
**24 to 59 months**	9130	17	5	22	**1.2**	2918	23	13	36	**6.2**	1686	9	**5.3**	**8.8**
**≥5 years**	144516	50	29	79	**0.3**	53583	83	45	128	**1.2**	17861	38	**2.1**	**3.5**
**Total**	**160257**	**96**	**47**	**143**	**0.5**	**58063**	**125**	**66**	**191**	**1.6**	**19547**	**47**	**2.4**	**4.0**

*: Two census data sets, collected 12 months apart were available. The mean of the two censuses is reported.

**: Surveillance did not include pregnant women and children <2 years of age.

***: Rates were corrected for direct protection from cholera vaccination.

The site in India consisted of legally registered urban slum areas (*bustees*) within administrative wards 29 and 30 in the city of Kolkata [9]. The area has a high population density and residents do not have sufficient water supply or sanitary facilities. A baseline census of the study population was done in early 2003 and was updated yearly. Surveillance was conducted from May 2003 to April 2005. The mid-year population of the study area was 58,063, based on two censuses, 12 months apart (Table 1). Surveillance included residents in the study site of all age groups who presented with diarrhea to any of the five project health outposts set-up in the field and two at the city's infectious diseases and children's hospitals, the main referral centers for diarrhea. Rectal swabs were brought to the study laboratory for isolation of *V. cholerae* O1 or O139.

In Mozambique, the study area was Esturro, an impoverished urban neighborhood (*bairro*) in the city of Beira. From December 2003 to January 2004, healthy, non-pregnant residents of Esturro who were two years of age or older were invited to participate in a mass-vaccination campaign using a 2-dose recombinant cholera toxin B subunit, killed whole-cell oral cholera vaccine [10]. A baseline census in 2003 enumerated a total population of 21,818 persons in Esturro of whom 1,177 were less than two years of age and an estimated 5% (or 1,091 residents) were excluded because of potential pregnancy, leaving a target population of 19,547 persons (Table 1). About 57% of the study population received 2 doses and 72% received the first dose of vaccine [10]. As part of a case-control study, surveillance was conducted from January to December 2004 and included Esturrro residents who presented with diarrhea to the Beira Cholera Treatment Center. Pregnant women and children under two years of age were excluded from the surveillance. Rectal swabs were brought to the study laboratory for isolation of *V. cholerae* O1 and O139. The case-control analysis showed adjusted ORs for vaccine protection of 0.16 from 2 doses and 0.22 from at least one dose of vaccine [10]. Thus, the incidence of cholera was corrected (not accounting for herd immunity) according to the following formula:
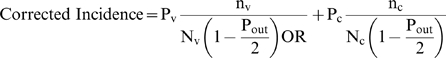
Where:

P_v_ is the proportion of the target population that is vaccinated (received at least 1 dose)

P_c_ is the proportion of the target population that is not vaccinated (received no vaccine)

n_v_ is the number of cholera cases detected among vaccinees during the first year

n_c_ is the number of cholera cases detected among non-vaccinees during the first year

N_v_ is the number at baseline who were vaccinated (received at least 1 dose)

N_c_ is the number at baseline who were not vaccinated (received no vaccine)

P_out_ is the proportion who outmigrated between baseline and one year (data not available)

OR is the odds ratio for vaccine protection (adjusted OR = 0.22)

### Data management

In all study sites, case report forms were double-entered into data entry programs using FoxPro software (Microsoft, Redmond, WA). The data management programs included checks for error and consistency. We estimated the annual incidence of cholera using population as the denominator and the age-specific number of cholera cases among the residents of the study area as the numerator. The cohort under surveillance was dynamic and included all cases with culture-confirmed cholera residing in the catchment area.

### Ethics

The surveillance was conducted following the principles governing biomedical research involving human subjects. In the Jakarta and Kolkata surveillance, verbal informed consent was obtained. This was considered as appropriate and sufficient since obtaining a history, physical examination, and stool specimen for culture of *V. cholerae* in cholera-endemic sites are part of good management of diarrhea patients [8],[9]. During the mass vaccination in Beira, written informed consent was obtained. During the case-control study in Beira that followed the mass vaccination, written informed consent was also obtained. Aside from a history, physical examination, and stool specimen for culture of *V. cholerae,* we asked each case for permission to visit his/her home (to recruit neighborhood controls) and we collected socio-behavioral data from the cases and controls [10]. The local ethics committees of each participating site, the WHO Secretariat Committee on Research Involving Human Subjects, and the International Vaccine Institute Institutional Review Board approved the study procedures and protocols.

## Results

**Figure 1:**
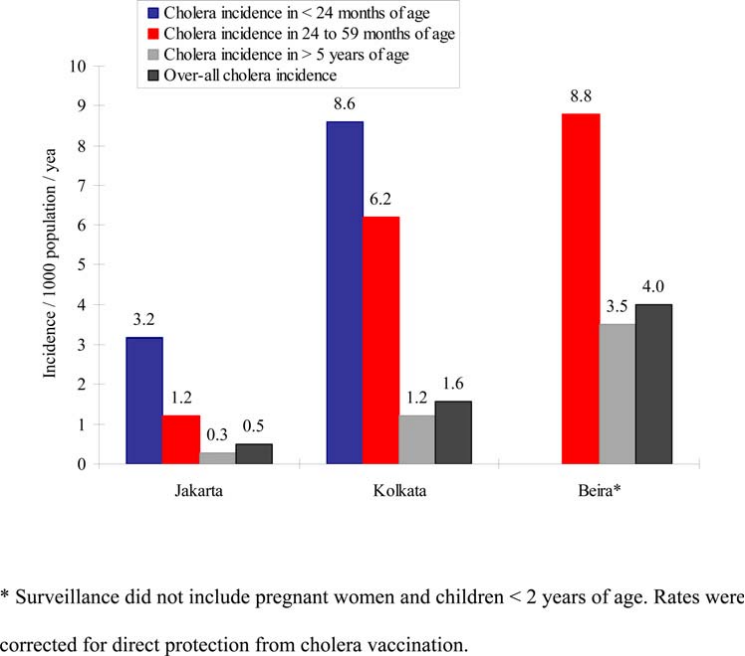
Cholera incidence (per 1000 population per year) in study sites in three countries.

We compared the annualized incidence (per 1,000 population) of cholera across the study sites (Figure 1 and Table 1). Overall rates ranged from 0.5 to 4.0 cases/1,000 population/year. The lowest overall rate was found in Jakarta, where the estimated incidence was 0.5/1,000/year. The incidence was three times higher in Kolkata (1.6/1000/year) and eight times higher in Beira (4.0/1000/year). The rates were highest in children under 5 years of age, with 8.8/1,000, 6.2/1,000, and 1.2/1000 among the 24 to 59 months old in Beira, Kolkata, and Jakarta, respectively. In the two sites where children under two years were also under observation (Jakarta and Kolkata), they were found to have even higher rates of cholera: 8.6/1,000 in Kolkata and 3.2/1,000 in Jakarta. Only *V. cholerae* O1 was isolated at all sites.

## Discussion

We found that young children bear the greatest burden of cholera. Cholera has traditionally been considered to occur infrequently in young children, and consequently, the WHO recommends that cholera should be suspected among those over two years of age who have acute watery diarrhea and severe dehydration if cholera is endemic in the local area [11]. Aside from our data, two other studies have shown that cholera is a significant problem in young children [6],[12], but neither provide population-based incidence. Our findings have implications for the enhanced benefit of cholera vaccination targeting specific age-groups in cholera-endemic areas. Protecting children against cholera may not only decrease the burden in this age group but decrease transmission of the disease to their family members and the community [13]. Immunization of adult women with killed oral cholera vaccines has been shown to confer herd immunity against cholera to children too young to be vaccinated [14].

Our comparison shows that the overall rates of cholera cases presenting for treatment varied widely across the study sites in three different countries, with the highest incidence in the African site. These findings add to the growing impression of the large cholera burden in Africa. In 2006, Africa reported 234,349 cholera cases to the WHO, accounting for 99% of the officially-notified global cholera [4]. Between 1995 and 2005, 66% of cholera outbreak reports to ProMed came from sub-Saharan Africa [15]. It has been suggested that the number of individuals at risk for cholera may be higher in Asia than in subSaharan Africa because of the higher population density in the former. But since cholera-endemic areas are likely to be more widespread in subSaharan Africa as evidenced by the officially reported cases [4], then the number of individuals at risk could potentially be higher in this continent.

Culture-confirmation showed that all isolates were *V. cholerae* O1. Previously, only *V. cholerae* serogroup O1 caused epidemic cholera. In late 1992, large outbreaks of cholera began in India and Bangladesh that were caused by a previously unrecognized serogroup of *V. cholerae*, designated O139, synonym Bengal. *V. cholerae* O1 had since been isolated in 11 countries in South-East Asia but less frequently in recent years. In 2006, 120 laboratory-confirmed *V. cholerae* O139 cases were reported from mainland China and 3 from Thailand but from no where else [4]. The expectation that *V. cholerae* O139 would become more widespread has apparently not occurred so far.

There are limitations to our data. First and foremost the surveillance sites were arbitrarily selected based on known cholera endemicity and may not be representative of the region. Although sentinel surveillance is the most reliable approach to collect comparable surveillance data between continents, data have to be interpreted with this limitation in mind. Second, there may have been variations in the intensity of surveillance despite similar case capture procedures across sites. Third, the size of the catchment population at each site differed considerably, ranging from 20,000 in Beira to 60,000 in Kolkata and 160,000 in Jakarta. Furthermore, the estimate in Beira had to be corrected for the direct protection from cholera vaccination. The true disease incidence may be even higher considering the herd (indirect) protection conferred by the vaccine [13]. Fourth, the data is based on study periods of one year in Beira and two years in Kolkata and Jakarta. Cholera incidence may vary over several years. It is possible that Beira was having an unusually high number of cases that year. Longer-term surveillance could provide additional important information but surveillance for long periods would be complicated by population mobility and consequent variations in the denominator. Fifth, we are reporting cholera incidence in areas with seasonal cholera, where the population is likely to have immunity from previous exposures. Attack rates during outbreaks at the time of natural disasters and complex emergencies occurring among populations without previous exposure to cholera are likely to be higher. Finally, passive surveillance in all three sites could only detect those cases which were perceived to be severe enough to require medical care and although we implemented methodology in each study site to optimize surveillance, some cases may have escaped our detection system. For example, health care utilization patterns may have influenced the relative differences in detected cholera between age groups. Young children with diarrhea may be more frequently taken for health care treatment compared to adults. It is possible that patients, particularly adults, escaped our detection system and that active surveillance could have identified more cases. However, it was not our intention to find all diarrhea cases in the community but only those perceived to be severe enough to require medical care and thus burdening the public health service.

This is to our knowledge the first comparison of the incidence of culture-confirmed treated cholera cases in endemic areas using standardized methods. In all our study sites, rates were highest in young children indicating the need to revisit the standard guidelines for clinically suspecting cholera. *V. cholerae* O139 was not detected in the study sites. We found considerable differences in the burden of disease in the three sites, with the African site having a multi-fold higher incidence compared to the Asian sites. Incidence of cholera is important when considering where and among whom interventions such as vaccination would be most beneficial.
